# Supplementary material for: A postzygotic GNA13 variant upregulates the RHOA/ROCK pathway and alters melanocyte function in a mosaic skin hypopigmentation syndrome
Source: Nat Commun. 2025 Feb 18;16:1751. doi: 10.1038/s41467-025-56995-4 (PMC11836271; doi:10.1038/s41467-025-56995-4)
Supplement: Supplementary file 1 — Supplementary Information [file 41467_2025_56995_MOESM1_ESM.pdf]

Supplementary information of the manuscript by El Masri *et al.* entitled "A postzygotic *GNA13* variant upregulates the RHOA/ROCK pathway and alters melanocyte function in a mosaic skin hypopigmentation syndrome"

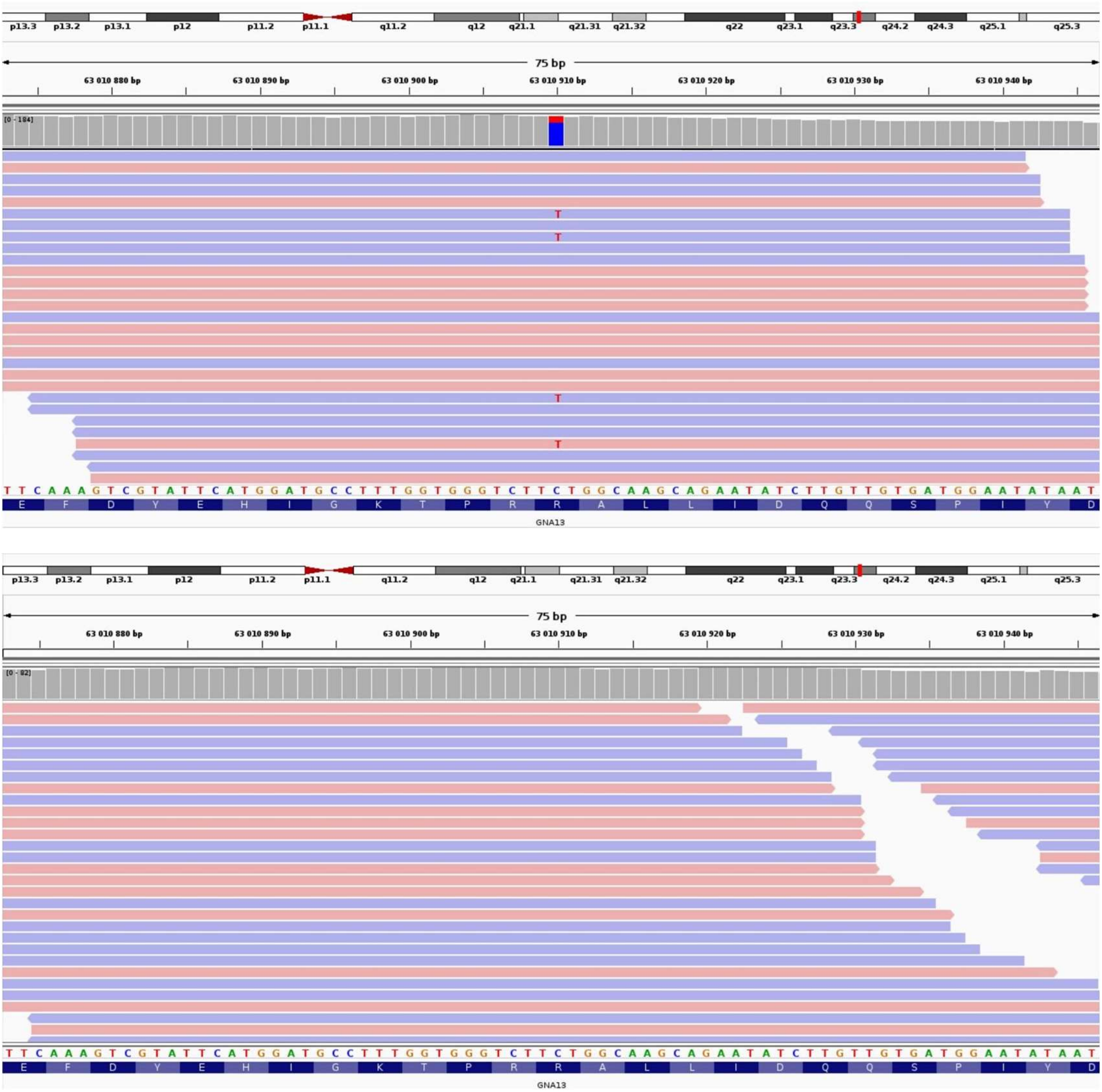

Supplementary Fig. 1: Integrative Genomics Viewer screenshots.

Top: screenshot of the postzygotic *GNA13* c.599G>A substitution (encoding p.(Arg200Lys)) in the affected skin of Patient 2. **Bottom**: absence of such substitution in the blood from this very same patient.

SK-MEL-28 cells

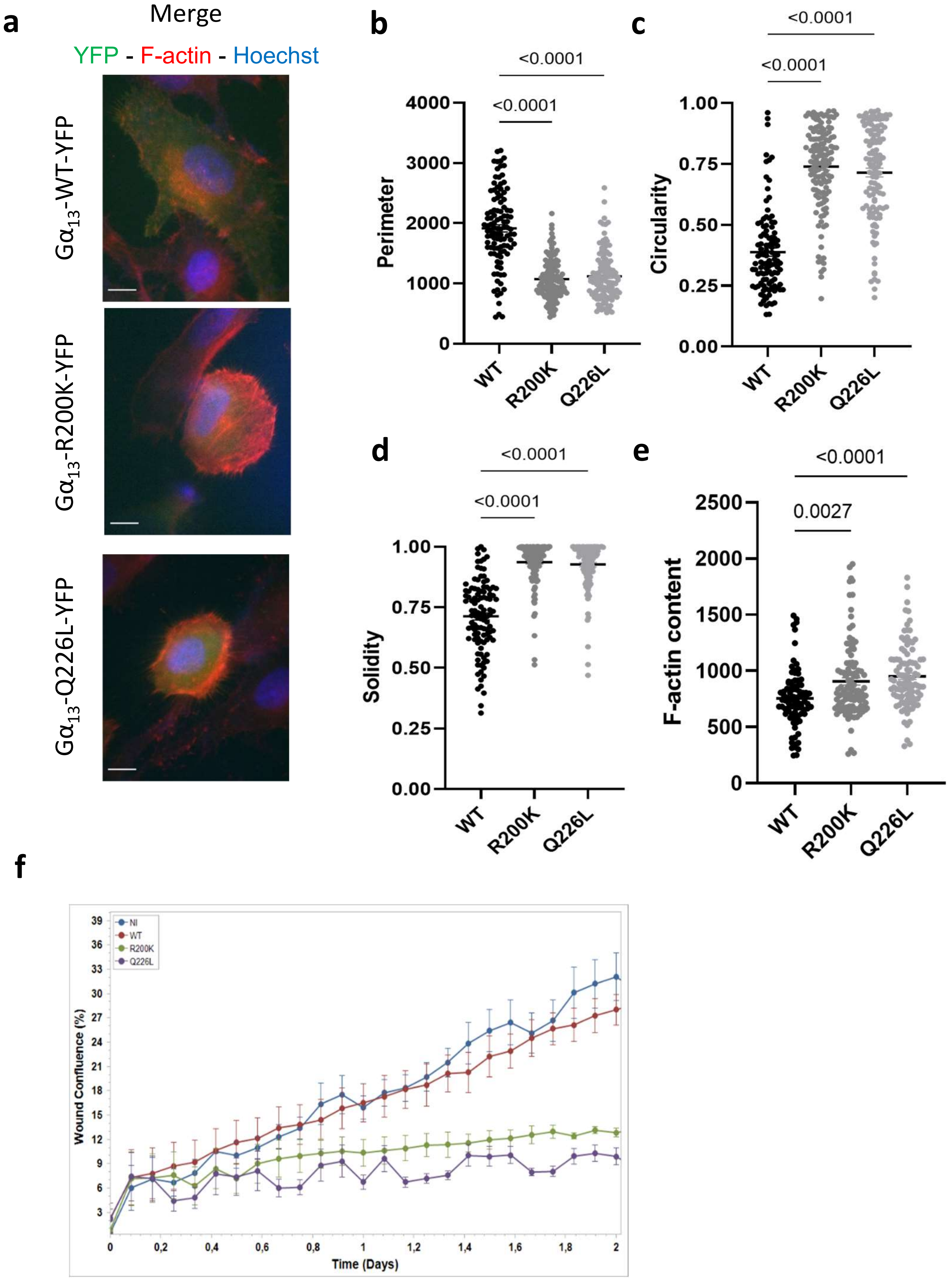

Supplementary Fig. 2: Effect of the Gα_{13} R200K mutation identified in patients on actin organisation, cell morphology and cell migration in SK-MEL-28 cells.

a Immunofluorescence images of Gα_{13} WT-YFP, Gα_{13} R200K-YFP or Gα_{13} Q226L-YFP (green) expressed in SK-MEL-28 cells. The cells were labelled for F-actin (red), and Hoechst for nuclei (blue). The scale bar is 10 µm. Quantification of cell morphology parameters including perimeter (**b**), circularity (**c**) and solidity (**d**); and of F-actin content (**e**) that corresponds to the intensity of the red signal. Means +/- SEM are shown. **f** Graphic representation of the migratory abilities of mock (blue), Gα_{13} WT-YFP (red), Gα_{13} R200K-YFP (green) and Gα_{13} Q226L-YFP (purple) expressed in SK-MEL-28 cells in the wound healing assay. The percentage of wound confluence was then quantified. Means +/- SEM are shown. ANOVA tests were performed. All graphs shown are representative of three independent experiments.

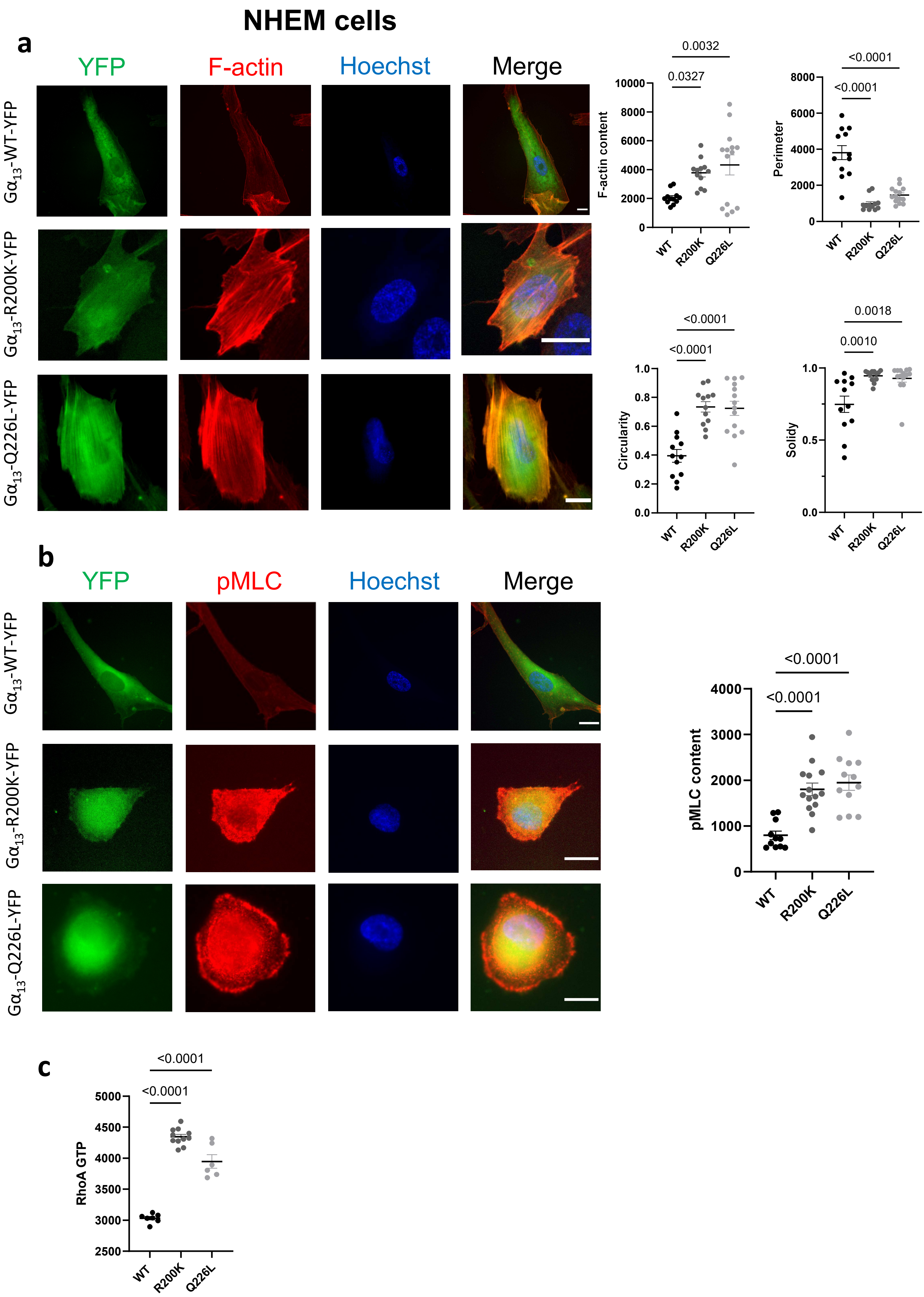

NHEM cells
a
YFP
F-actin
Hoechst
Merge
Gα13-WT-YFP
Gα13-R200K-YFP
Gα13-Q226L-YFP
0.0032
0.0327
F-actin content
<0.0001
<0.0001
Perimeter
<0.0001
<0.0001
Circularity
0.0018
0.0010
Solidity
WT
R200K
Q226L
b
YFP
pMLC
Hoechst
Merge
<0.0001
<0.0001
pMLC content
c
<0.0001
<0.0001
RhoA GTP

Supplementary Fig. 3: Effect of the Gα_{13} R200K mutation on the morphology, cytoskeleton and RHOA activity of primary Normal Human Epidermal Melanocytes (NHEM).

a,b Immunofluorescence images of Gα_{13} WT-YFP, Gα_{13} R200K-YFP or Gα_{13} Q226L-YFP (green) expressed in NHEM. Left panels: The cells were labelled with phalloidin for visualising F-actin (**a**) or pMLC (**b**) (red), and Hoechst for nuclei (blue). Right panels: Quantifications of morphological parameters (perimeter, circularity and solidity) and F-actin content (**a**), and pMLC levels (**b**). **c** Quantification of RHOA-GTP levels in Gα_{13} WT-YFP, Gα_{13} R200K-YFP or Gα_{13} Q226L-YFP-expressing NHEM cells. Means +/- SEM are shown.

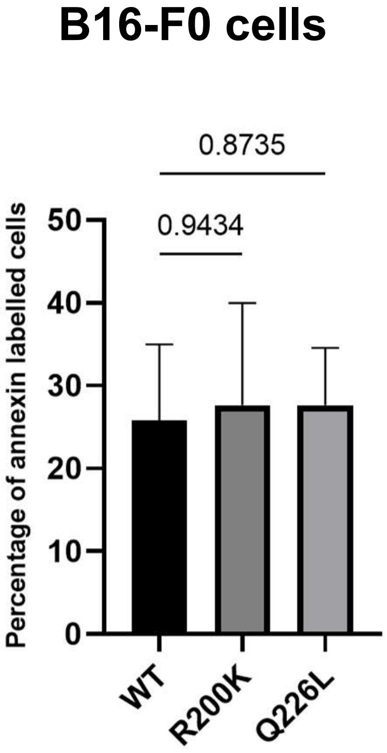

Supplementary Fig. 4: Effect of the Gα_{13} R200K mutation identified in patients on cell death.

Gα_{13} WT-YFP, Gα_{13} R200K-YFP or Gα_{13} Q226L-YFP expressing B16-F0 cells were labelled with Annexin V three days after being transfected, analysed by flow cytometry, and the percentage of Annexin V -positive cells was quantified. Means +/- SEM from four independent experiments are shown. ANOVA test was performed.

B16-F0 cells

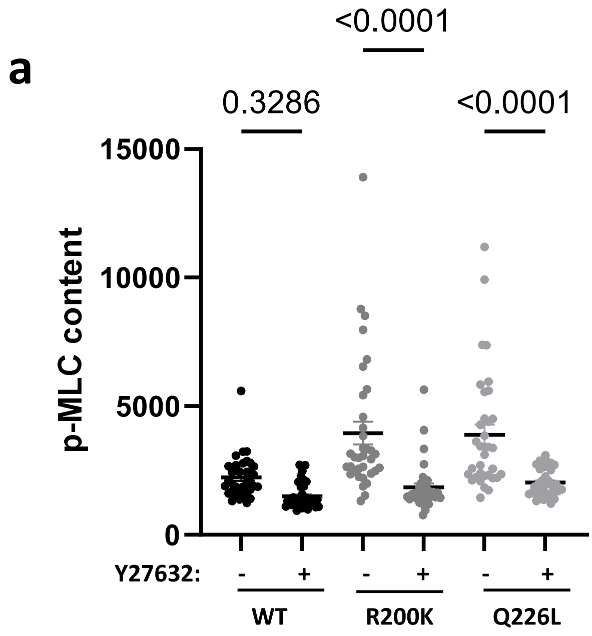

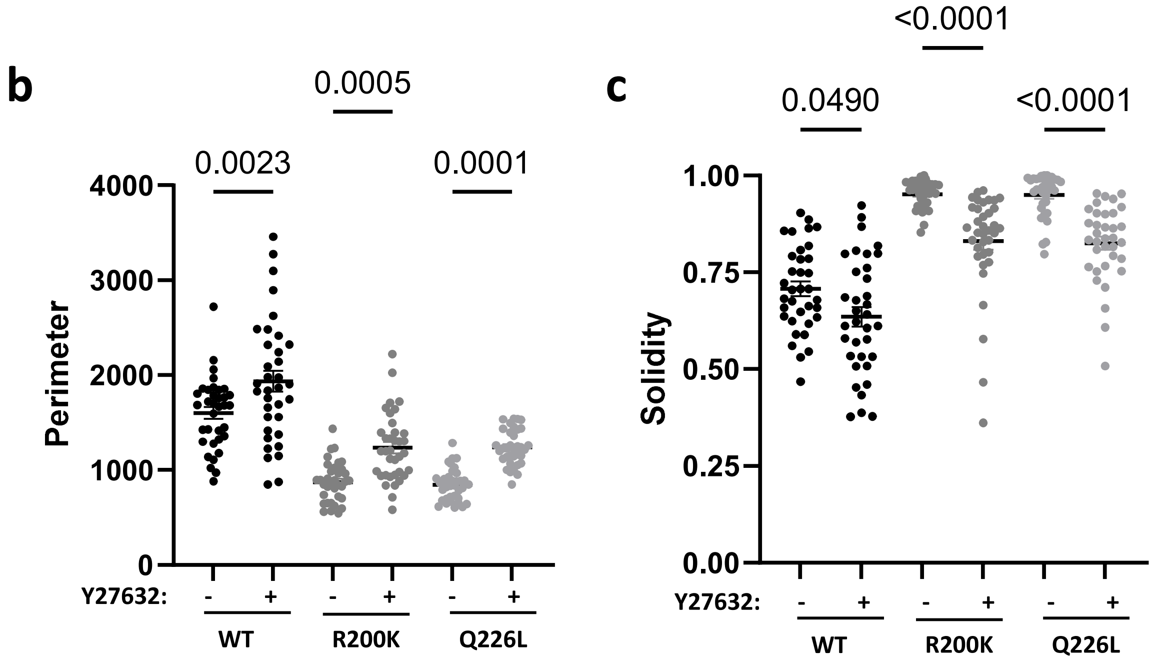

Supplementary Fig. 5: Effect of a ROCK inhibitor on pMLC content and cell morphology.

Quantification of pMLC content (**a**), perimeter (**b**) and solidity (**c**) in Gα_{13} WT-YFP, Gα_{13} R200K-YFP or Gα_{13} Q226L-YFP expressing B16-F0 cells, upon ROCK inhibition by Y27632 treatment. Means +/- SEM are shown. ANOVA tests were performed. All graphs shown are representative of three independent experiments.

NHEM cells

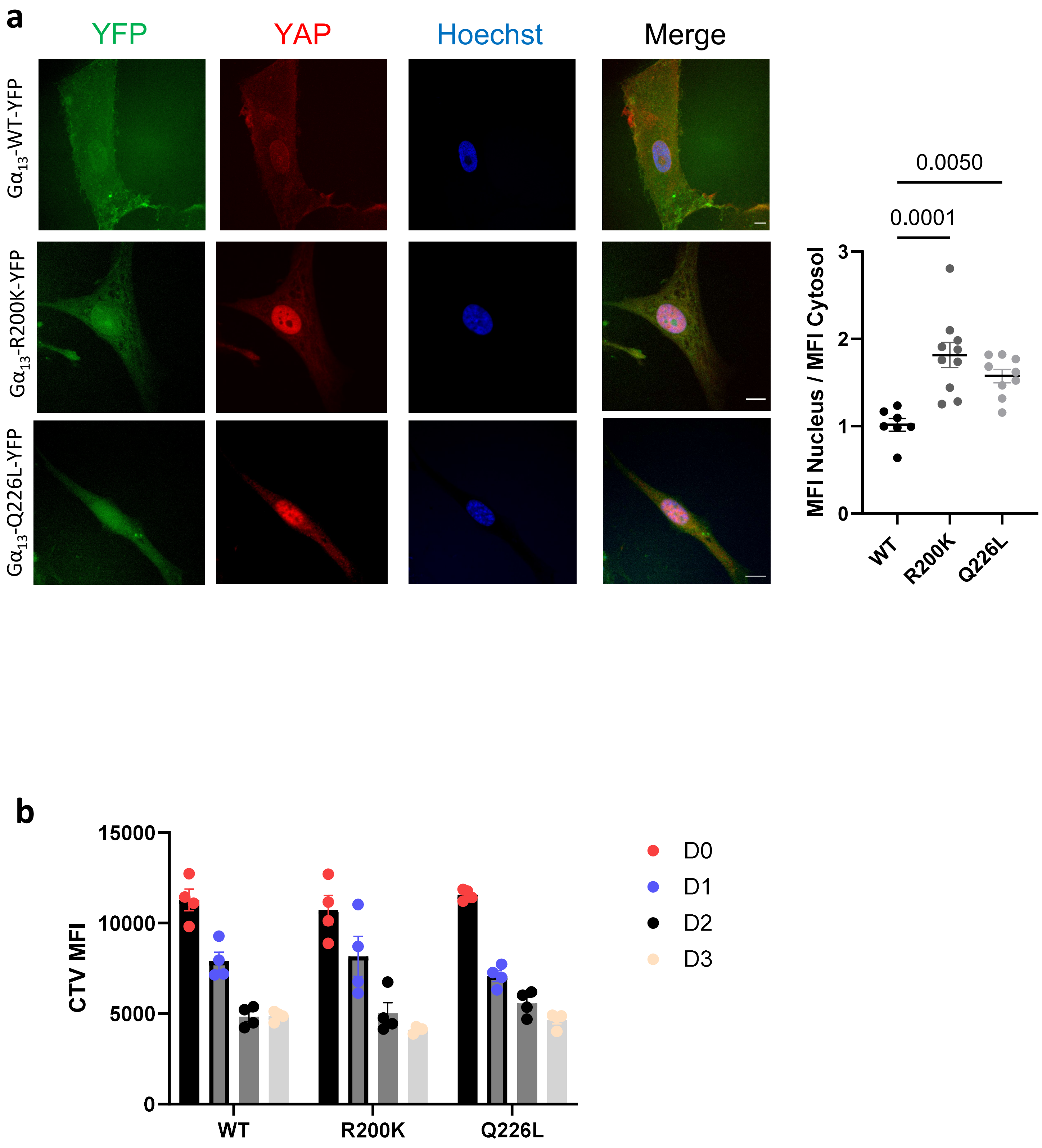
a
YFP
YAP
Hoechst
Merge
Gα13-WT-YFP
Gα13-R200K-YFP
Gα13-Q226L-YFP
MFI Nucleus / MFI Cytosol
0.0050
0.0001
0
1
2
3
WT
R200K
Q226L
b
CTV MFI
15000
10000
5000
0
WT
R200K
Q226L
D0
D1
D2
D3

Supplementary Fig. 6: Effect of the Gα_{13} R200K mutation on YAP signalling in NHEM.

a. Left: Immunofluorescence images of Gα_{13} WT-YFP, Gα_{13} R200K-YFP or Gα_{13} Q226L-YFP (green), YAP (red) and the nucleus (Hoechst, blue) in NHEM. Right: Single cell quantification of the ratio of YAP mean fluorescence intensity (MFI) in the nucleus over the cytosol. Means +/- SEM are shown. **b**. Flow cytometry quantification of the CellTrace violet mean fluorescence intensity (CTV MFI) at days 0, 1, 2 and 3 following CTV staining of NHEM. The dye dilution within days traces the multiple generations of the proliferating cells.

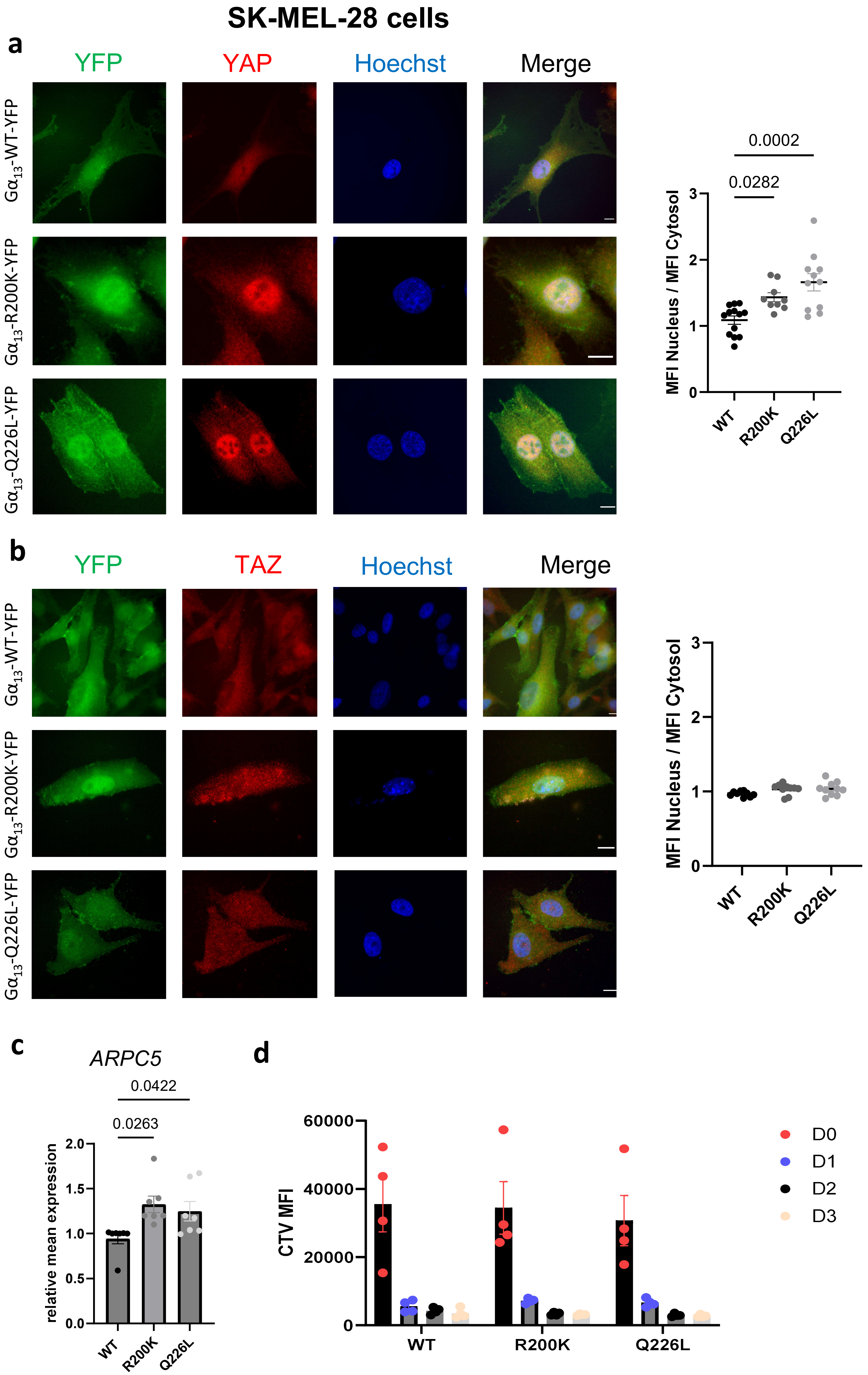
SK-MEL-28 cells
a
YFP
YAP
Hoechst
Merge
Gα13-WT-YFP
Gα13-R200K-YFP
Gα13-Q226L-YFP
0.0002
0.0282
MFI Nucleus / MFI Cytosol
0
1
2
3
WT
R200K
Q226L
b
YFP
TAZ
Hoechst
Merge
Gα13-WT-YFP
Gα13-R200K-YFP
Gα13-Q226L-YFP
MFI Nucleus / MFI Cytosol
0
1
2
3
WT
R200K
Q226L
c
ARPC5
0.0422
0.0263
relative mean expression
0.0
0.5
1.0
1.5
2.0
WT
R200K
Q226L
d
CTV MFI
0
20000
40000
60000
WT
R200K
Q226L
D0
D1
D2
D3

Supplementary Fig. 7: Effect of the Gα_{13} R200K mutation on YAP signalling in SK-MEL-28.

a,b Left: Immunofluorescence images of Gα_{13} WT-YFP, Gα_{13} R200K-YFP or Gα_{13} Q226L-YFP (green), YAP (**a**) or TAZ (**b**) (red) and the nucleus (Hoechst, blue) in SK-MEL-28. Right: Single cell quantification of the ratio of YAP (**a**) or TAZ (**b**) mean fluorescence intensity (MFI) in the nucleus over the cytosol. **c** Quantification of the mRNA expression levels of ARPC5 normalised to GAPDH expression in SK-MEL-28 cells expressing Gα_{13} WT-YFP, Gα_{13} R200K-YFP or Gα_{13} Q226L-YFP. Means +/- SEM from seven independent experiments are shown. **d.** Flow cytometry quantification of the CellTrace violet mean fluorescence intensity (CTV MFI) at days 0, 1, 2 and 3 following CTV staining of SK-MEL28. The dye dilution within days traces the multiple generations of the proliferating cells.

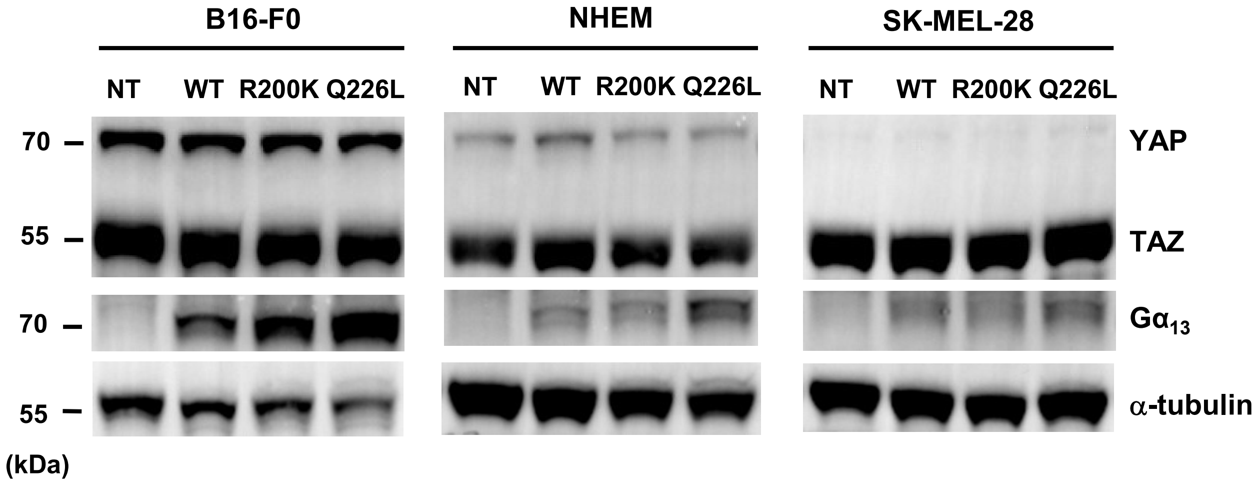

Supplementary Fig. 8: Effect of the Gα$_{13}$ R200K mutation on YAP and TAZ expression.

Western blot analysis of YAP and TAZ expression in Gα$_{13}$ WT, R200K and Q226L expressing B16-F0, NHEM and SK-MEL28 cells.

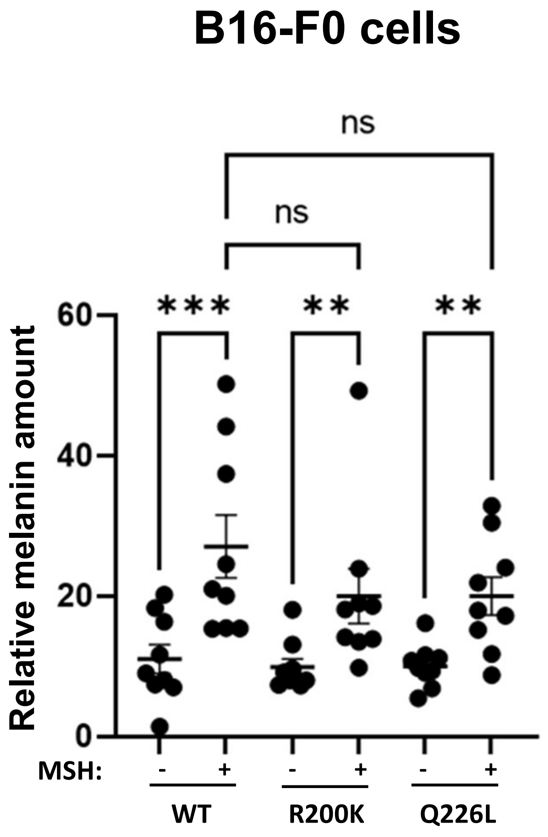

Supplementary Fig. 9: Effect of the Gα$_{13}$ R200K mutation identified in patients on melanin production.

Gα$_{13}$ WT-YFP, Gα$_{13}$ R200K-YFP and Gα$_{13}$ Q226L-YFP expressing B16-F0 cells were stimulated with MSH for 48 hours, lysed and the melanin content was measured. The relative melanin amount was normalised for 10^5 cells. Means +/- SEM from nine independent experiments are shown. Friedman test was performed.

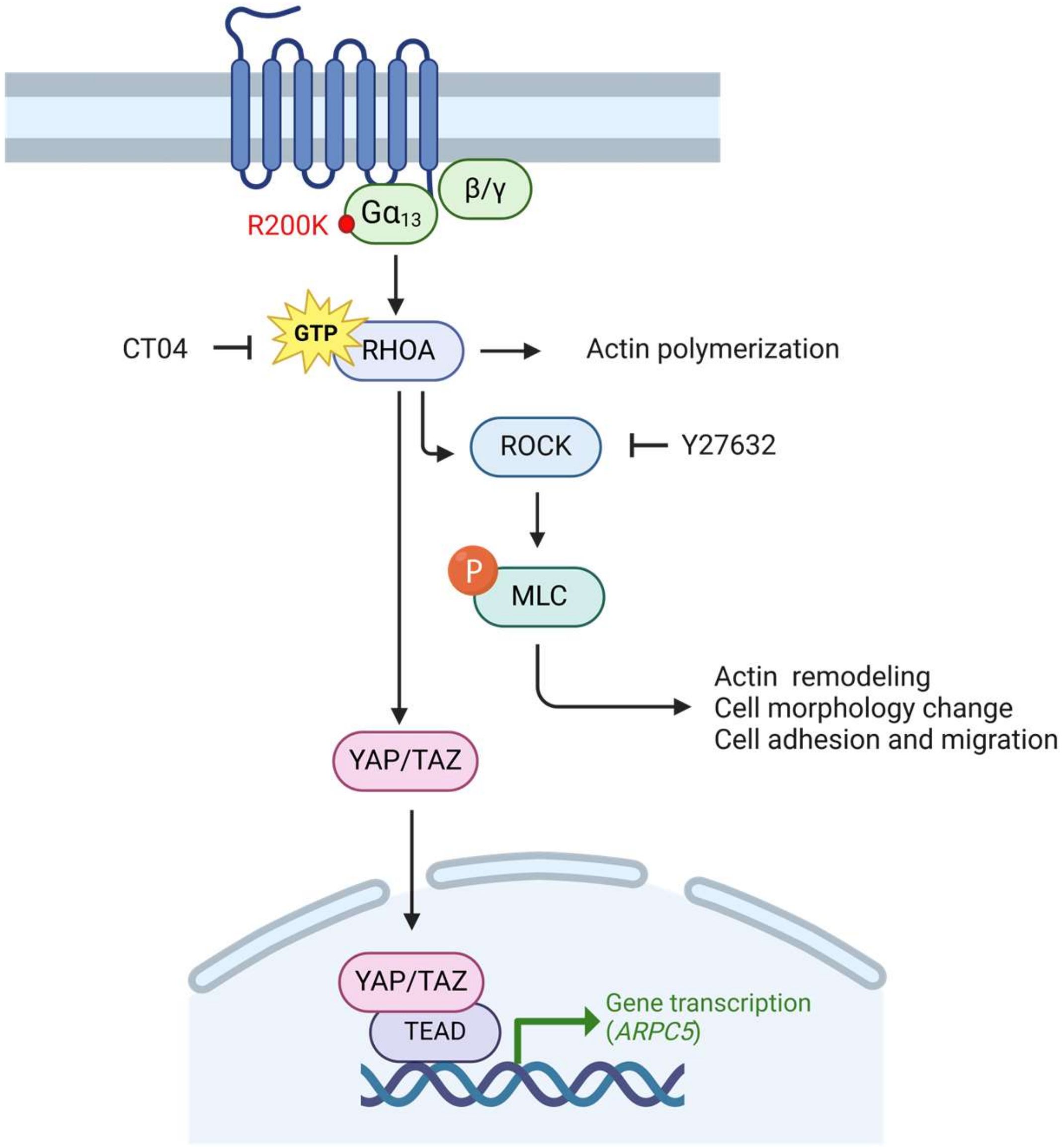

Supplementary Fig. 10:

Schematic showing the effects of the Gα_{13} R200K mutation on the activation of the RHOA/ROCK/MLC pathway and the YAP/TAZ nuclear translocation. Created in BioRender. Masri, R. (2025) https://BioRender.com/m10i137.
